# Quality Improvement Attitudes among Saudi Nurses in Hospitals in Qassim, Saudi Arabia: A Cross-Sectional Survey

**DOI:** 10.3390/healthcare11010049

**Published:** 2022-12-24

**Authors:** Ibrahim Alasqah, Muteb Alotaibi, Cris Adolfo, Mahmudul Hassan Al Imam, Bader Alrasheadi, Adel Alhindi, Hassan Altakroni, Ilias Mahmud

**Affiliations:** 1 Department of Public Health, College of Public Health and Health Informatics, Qassim University, Al Bukairiyah 52741, Saudi Arabia; 2 Department of Nursing, College of Applied Medical Sciences, Majmaah University, Majmaah 11952, Saudi Arabia; 3 Nursing Administration, Dariyah General Hospital, Dariyah 58760, Saudi Arabia; 4 School of Health, Medical and Applied Sciences, Central Queensland University, Rockhampton, QLD 4701, Australia; 5 Central Queensland Public Health Unit, Central Queensland Hospital and Health Service, Rockhampton, QLD 4700, Australia; 6 Nursing Administration, Ministry of Health, Buraidah 52384, Saudi Arabia; 7 College of Applied Medical Sciences, Buraidah Colleges, Buraydah 51452, Saudi Arabia

**Keywords:** nurse, quality improvement, healthcare, Saudi Arabia

## Abstract

Background: This study aimed to provide an overview of perceptions of quality improvement among nurses working in Saudi Arabia. Methods: We conducted a descriptive cross-sectional study of 497 Saudi nurses working in public and private hospitals in Al-Qassim Province. Descriptive statistics were computed for quality improvement nursing attitude items and demographic factors. Results: A total of 497 nurses took part in the study; 29.1% of participants were females, and half of the participants were between the ages of 25–30 years. Most respondents were employed in governmental hospitals (98.7%), and 41.9% of participants had work experience ranging between 1 and 5 years. Nurses involved in providing direct patient care recognize the quality improvement attitudes related to changes in the healthcare delivery processes. Saudi nurses’ quality improvement nursing attitudes were moderate. Female, married, and older age group nurses and nurses who were working fewer hours per week showed better quality improvement attitudes. Conclusions: Saudi nurses’ quality improvement nursing attitudes are found to be moderate. Age, gender, marital status, and working hours of nurses are associated with their quality improvement attitudes. To empower nurses to improve healthcare, nursing administrators need to focus on improving the quality improvement attitudes environment.

## 1. Background 

In recent decades, the importance of and attention given to quality improvement (QI) in hospitals has increased internationally. In health care, QI is a widely used systematic framework that improves the quality of the patient care delivered by health care professionals [1]. QI involves unceasing efforts to achieve steady and established process results for both patients and the healthcare institution in question [1]. Implementing QI in a healthcare organization has several benefits, such as the promotion of patient progress, decreased morbidity and mortality, effective and efficient managerial processes, and decreased medical errors and failures [1]. In healthcare systems, QI is also used as a tool to improve the quality of healthcare [2]. Healthcare workers play a key role in refining the quality of care in hospitals [3]. Nurses, like other professionals, play a crucial role in improving the quality of healthcare services since they are the primary providers of patient care and serve as members of the healthcare staff who are concerned with patient safety [4], which might be the reason nurses play a significant role in improving overall hospital quality.

In Saudi Arabia, the development of the healthcare system by capable and well-secured healthcare providers, including nurses, has become the focus of the Ministry of Health [1]. The national policy aimed at the Saudization of the workforce has played a significant role in increasing recognition of nursing as a suitable vocation for Saudi men and women and has improved and increased the demand for newly qualified Saudi registered nurses with bachelor’s degrees [1]. In the Middle East, this policy is related to the likelihood of increasing the number of indigenous nationals who are registered nurses [5]. Moreover, Saudi nurses must be qualified, based on their capabilities, to provide care services in each situation based on certain standards of nursing care; so, healthcare quality can be described as the attempt to ensure excellent care standards [6].

Extensive research has shown that the provision of basic data on nurses’ perceptions of QI could improve the quality of healthcare. It has also been widely acknowledged that nurses’ active involvement is vital for QI in any organizational setting [3]. Globally, research on the QI competencies of nurses suggests that nurses are often not prepared by the academic programs; they often do not look for or receive adequate learning opportunities to build their QI competence [7]. The negative perception of nurses toward QI has significant implications for their voluntary professional development activities since they may not want to develop in areas they do not perceive as valuable. Furthermore, regulatory incentive following nursing graduation on QI is also lacking [7]. 

Without nurses, being frontline staff with adequate QI knowledge and attitudes, patient safety cannot improve sufficiently. However, QI initiatives among Saudi nurses have not usually obtained the full engagement of nurses due to recent changes to QI policy [3]. While several hospitals have considered the role of nurses in QI to be crucial, there is still a great deal of uncertainty in the relationship between this situation and the QI attitudes of Saudi nurses. To date, no consensus has been reached about the attitudes of Saudi nurses concerning QI. Due to the importance of nurses’ attitudes in determining QI, this study assessed these attitudes. This study aimed to provide an overview of perceptions of quality improvement among nurses working in Saudi Arabia and determine the factors associated with it. This study provides a comprehensive understanding of the role of Saudi nurses in QI and the challenges they face; it also offers significant insights into how hospitals can enhance their use of human resources to improve the quality of care, Saudi nurses’ empowerment, and QI initiatives in the future. 

## 2. Methods

This study employed a descriptive cross-sectional design to investigate the perceptions of quality improvement attitudes shown by Saudi nurses working in Saudi Arabia. Convenience sampling was used in the study in which nurses were invited by email to take part in the study. An invitation email was sent to 497 Saudi nurses working in public and private hospitals in Al-Qassim Province. However, only 497 of 696 nurses were accepted and took part in the study, with a response rate of 71.40%. Male or female Saudi nurses who provided direct patient care or who were outpatient department nurses, i.e., those who had been employed as a nurse in Saudi Arabia, voluntarily consented to take part. Unit managers and clinical resource nurses were excluded because they handled policies, systems, procedures, and organizational climates. These professionals supervised staff nurses and were not directly involved in patient care. Moreover, they engaged in different forms of preparation for work and different types of training. An authorization letter to conduct the research was issued and signed by the supervisor and was duly approved by the head of the department. Subsequently, this letter was presented to the chief nurse of each hospital to obtain permission to conduct the study. Similarly, the researcher sought permission to find nursing staff who could take part in the study.

The Quality Improvement Nursing Attitude (QINA) scale was used to assess the quality improvement attitudes of Saudi nurses. Strong support for the internal consistency reliability and face validity of the Quality Improvement Nursing Attitude Scale was found in an earlier study [8]. All members of the nursing staff were informed about the questionnaire, and the research purpose, assured that participation was voluntary and that their identities would not be revealed, and asked whether they were interested in taking part. Written consent was obtained from each participant, and the questionnaire was later completed during working hours. A pilot test measured the correctness of the research measures and inquiries used in the study. The pilot test analyzed the questions presented on the questionnaire. The pilot test was distributed to 35 Saudi nurses working in the different hospitals included in the study. These 35 responses were representative of the larger group. The pilot test requested feedback and responses concerning the duration required to complete the survey and whether the survey included any unclear questions. This feedback helped with the deletion of unclear questions, regulated the satisfactory range of responses, ensured that the responses could be understood as projected and that all queries would receive responses, helped the rewording of questions that received unforeseen responses, and allowed the survey to be shortened, reviewed, and subjected to future pilot testing. Before each interview, the researchers gave the respondent a short explanatory introduction, clarified the terms used in the questionnaire, and answered any questions. After completing the questionnaire, respondents were asked to give it to the researchers. The data were then counted, computed, analyzed, and interpreted. Data were collected using Google Forms and entered into SPSS (Statistical Package for the Social Sciences) version 23.0 software for data analyses. The validity and reliability were tested using the internal consistency method based on Pearson correlations among the items and the total degree of the scale; Cronbach’s alpha test was used to test the reliability. The Shapiro–Wilk test was performed to assess the normality of the data. The frequencies, percentages, means, and standard deviations were computed for Quality Improvement Nursing Attitude (QINA) items and demographic factors. Independent sample *t*-tests were performed to assess the association between the outcome variable and the independent variables.

## 3. Results

A total of 497 healthcare staff members took part in the current study, including 348 (70.9%) males and 143 (29.1%) females. Half of the respondents were aged between 25–30 years old, and 313 (63.6%) held a bachelor’s degree. A total of 202 (41.9%) participants had between 1 and 5 years of experience. The participants’ sociodemographic characteristics can be found in Table 1. 

There was a significantly low but positive relationship between gender and QINA (*r* = 0.133, *p* < 0.05), as female nurses were more likely to show higher QINA than male nurses. Further, married nurses showed higher QINA scores (*X^2^* = 21.90, *p* < 0.05) than single nurses. In terms of age, older nurses reported higher QINA compared to younger nurses (*r* = 0.146, *p* < 0.05). Moreover, the fewer hours worked by a nurse per week, the more that nurse exhibits QINA (*r* = −0.109, *p* < 0.05). 

### 3.1. Descriptive Analysis of the Quality Improvement Nursing Attitude (QINA)

As shown in Table 2, the QINA scale measures nursing attitudes in terms of the value of quality competencies, nurses’ beliefs of their role in quality improvement, and the organizational culture in which they practice. Items in this scale are scored on a 5-point Likert scale (ranging from strongly agree to strongly disagree). In this study, the mean score varied from (*M* = 3.08, *SD* = 1.30) to (*M* = 3.30, *SD* = 1.25), and the overall score was (*M* = 3.22, *SD* = 1.08). Therefore, the QI of nurses was indicated to be moderate.

### 3.2. The Association between the Quality Improvement Nursing Attitude (Qina) Scale and Demographic Factors

As shown in Table 3, three tests were used to define the relationship between demographic factors and the QINA scale. An independent *t*-test was conducted to compare the QINA scores between males and females. There were significant differences in scores with advantages between females (*M* = 3.49, *SD* = 0.90) and males (*M* = 3.10, *SD* = 1.14) (*t* = 3.60, *p* < 0.05).

## 4. Discussion

The aim of this study was to provide an overview of perceptions of quality improvement among nurses working in Saudi Arabia. Our study found that nurses who performed direct services are in an outstanding position to recognize the need for QI-related changes to healthcare delivery processes. Saudi nurses’ attitudes towards QI were moderate. Female nurses showed better quality improvement attitudes than males. Married nurses showed better quality improvement attitudes than single nurses. The older the nurses were, the more often they practiced quality improvement in the hospital. The fewer hours nurses worked per week, the more they showed quality improvement attitudes.

Furthermore, there was a significantly low but positive relationship between gender and QINA, as females were more likely to exhibit QINA than men. It is noteworthy that the achievement of gender diversity in nursing is the next step in providing quality care. These results agree with the conclusions of earlier studies, showing that female nurses engage in more nurturing care than males [9]. According to Teunissen, Rotink, and Lagro-Janssen (2016), male nurses show more hegemonic masculinity than women [10]. This study also found that male nurses perceived that they had more power than females. However, earlier studies have noted that males described themselves as softer, more emotional, and more modern than females [11]. This feminine emotion may result in an increase in quality standards among men [9]. The results of this also support this statement.

This study found a significant relationship between marital status and QINA, and the results of the crosstab analysis showed that married nurses exhibited more QINA than others. These results affirmed the conclusions of an earlier study, showing that nurses being married is a predictor of exhibiting QINA, while nurses being divorced is a predictor of lower QINA [12]. The marital status variable is related to beliefs of social support because married individuals receive more social support from their families, co-workers, or spiritual beliefs than single individuals. According to Buchanan, Dawkins, and Lindo (2015), the reason for this difference is that married nurses obtain better emotional support from their spouses, which decreases their stress and can thus enhance quality standards [13]. Therefore, healthcare administrators should develop strategies for improving QINA among single nurses to ensure that nurses can satisfy better care quality standards.

There was a significantly low and positive relationship between age and QINA; that is, the older the nurses were, the more QINA they showed. This finding is consistent with the results of an earlier study showing that the older the individual is, the greater the individual’s levels of maturity and professional experience [14]. Kramer, Brewer, and Maguire (2013) [15] reported that older nurses effectively practice in an environment of quality care due to their greater exposure to and experience with healthcare accreditation. Older nurses are more flexible and show superior scheduling, education, and training; they also play alternative or redesigned roles. Therefore, this situation justifies the need for continued professional development activities with a greater focus on newer and younger nurses. Justifying the need to provide continuing education and training to younger nurses can open educational opportunities, which may, in turn, lead to an increase in employee engagement with quality nurses.

There was a significantly low and negative relationship between the number of hours worked per week and QINA; that is, the fewer hours a nurse works per week, the more QINA that nurse exhibits. This finding is noteworthy due to the increasing concern about the impacts of nurses’ long working hours despite the increasing capabilities of healthcare systems [16]. This result is congruent with the conclusions of another earlier study that reported that longer working hours can shorten nurses’ rest periods between shifts, thus resulting in higher risks of burnout and harm to their overall health status [17]. A longer working period is related to the occurrence of medication errors, insufficient sleep quality, and decreased well-being [18]. However, other countries, such as Korea, have noted that overtime is common among nurses in university hospitals [19]. Therefore, the nursing administration must adjust nurses’ schedules and create various approaches to the task of solving this problem, such as hiring more nurses in healthcare facilities.

The statement “I value technologies that support clinical decision-making, error prevention, and care coordination” is associated with the highest mean score, while the statement “I value my contributions to the positive quality outcomes of care in local care settings” is associated with the lowest mean score. The mean scores varied from 3.08 (*SD* = 1.30) to 3.30 (*SD* = 1.25), and the overall mean score was 3.22 (*SD* = 1.08). Therefore, the results of this study indicated that nurses have moderate QINA. This finding is consistent with the literature, showing that QI implementation is more complex and has many more important components that are necessary for the development of effective QI interventions and strategies [20]. Therefore, it is necessary to develop more interventions designed to improve QI. According to Wells et al. (2018) [21], nurses who provide direct services are in an outstanding position to recognize the need for QI change in healthcare delivery processes. In this regard, nurses must think and behave in distinctive ways since this context merits improvement programs. By empowering nurses’ efforts to improve their health care, nursing administrators must also take part in reshaping the QI environment. 

There are some limitations associated with this study. Our study findings are based on the data collected from conveniently recruited samples, which might limit the generalization of the study findings. Further, this study utilized self-reported data, and therefore, we cannot rule out the possibility of information bias. Future studies incorporating observational data and documentary analysis are essential. Additionally, our study findings might not be generalized to other institutes. Despite these limitations, our study reports perceptions of quality improvement attitudes shown by Saudi nurses working in Saudi Arabia.

## 5. Conclusions

This is the very first study of its kind which assessed the quality improvement attitudes of Saudi nurses. This study found that the quality improvement attitudes of Saudi nurses were moderate. Female nurses showed better quality improvement attitudes than male nurses. Married nurses showed better quality improvement attitudes than single nurses. The older the nurses were, the more they showed quality improvement in the hospital. The fewer the number of hours nurses worked per week, the more they showed quality improvement attitudes. QI in the context of healthcare services could be reached as the result of positive interactions among different departments that can collaborate to construct a dynamic mechanism to continually improve the outcomes of healthcare services.

## Figures and Tables

**Table 1:** Demographic factors of nurses (*n* = 497).

Factor		Frequency	Percent
Gender	Male	348	70.9%
	Female	143	29.1%
Marital status	Single	137	27.8%
	Married	297	60.4%
	Separated	49	10.0%
	Widow	9	1.8%
Age	25–30 years old	235	48.1%
	31–35 years old	179	36.6%
	More than 35 years old	75	15.3%
Level of education	Diploma	134	27.2%
	Bachelor’s degree	313	63.6%
	Other	45	9.1%
Experience	1–5 years	202	41.9%
	6–10 years	194	40.2%
	More than 10 years	86	17.8%
Hospital type	Governmental hospital	448	98.7%
	Private hospital	6	1.3%
Area of practice	Emergency Department	91	20.5%
	Outpatient Department	50	11.3%
	Medical Department	81	18.3%
	Surgical Department	74	16.7%
	ICU	70	15.8%
	Artificial Kidney Unit	34	7.7%
	Other	43	9.7%
Hours worked per week	40–45 hours	256	56.85
	46–50 hours	165	36.6%
	More than 50 hours	30	6.7%

**Table 2:** Descriptive analysis of the Quality Improvement Nursing Attitude scale (*n* = 497).

No.	Statements	Mean	*SD*
1	I value my contributions to the positive quality outcomes of care in local care settings.	3.01	1.34
2	I enjoy being part of the change in my unit to improve the quality of care.	3.11	1.35
3	I have value in institutional efforts to improve care.	3.08	1.30
4	I believe that consistent deviation from standards of care negatively affects the quality of care.	3.09	1.28
5	Good patient care depends on the use of tools that measure quality improvement.	3.18	1.29
6	Continuous quality improvement is an essential part of the daily work of the bedside nurse.	3.22	1.26
7	I value active partnerships with patients in the planning, implementation, and evaluation of care.	3.18	1.26
8	I respect and encourage the individual expression of patients’ values, preferences, and expressed needs in caring for my patients.	3.17	1.25
9	I value how research contributes to my practice by supplying evidence of best practices.	3.18	1.27
10	I believe I should take part in structuring the work environment to help integration of new evidence into practice standards.	3.18	1.30
11	I value technologies that support clinical decision-making, error prevention, and care coordination.	3.26	1.26
12	I believe I should be involved in the design, selection, and use of information technologies to support patient care.	3.21	1.29
13	I play a role in analysing unsafe practices, errors, and designing system improvements.	3.19	1.28
14	Quality outcomes are dependent on the following: my acceptance of patient contributions to care, correct use of electronic medical records, nursing research, and ongoing collaboration with team members.	3.25	1.27
15	I believe that I should be able to effectively communicate with all members of the healthcare team to provide qualified care.	3.24	1.27
16	I respect other healthcare team members’ perspectives and ability in making decisions about patient care.	3.26	1.29
17	When I plan for caring for my patient, I believe that best practices, patient preferences, and interdisciplinary contributions are essential to safety and quality care.	3.26	1.29
18	When evaluating safety risks to my patient, I consider all the following: input from the patient, family members, and other health care professionals; documented information in the electronic medical record; and current evidence.	3.25	1.27
19	Technology and the electronic medical record give me the opportunity to collaborate with other nurses and healthcare professionals to achieve safe and quality outcomes for my patients.	3.29	1.29
20	When I see a risk that could compromise the safety of my patient, I immediately figure out whether this risk is a system-wide problem.	3.28	1.26
21	I believe that nurses should not deviate from best practices to save time or effort spent on work.	3.26	1.32
22	I often look to examine patient preferences and current research to guide me in my efforts to reduce patient harm or enhance quality outcomes.	3.29	1.26
23	When I see other nurses deviating from the standard of care, I feel powerless to influence their practice.	3.17	1.28
24	I believe that my managers value information regarding the work habits that affect the quality of care in my unit.	3.27	1.28
25	I believe that issues and problems involving patient safety and quality care are addressed by my unit manager or another leader promptly.	3.24	1.26
26	I am involved in the quality improvement process perfectly.	3.31	1.26
27	I feel that issues with patient safety are ‘systematic problems’ by my managers.	3.20	1.26
28	When patient safety is compromised, this situation is reliably reported.	3.29	1.28
29	We have a ‘culture of safety in my unit.	3.27	1.27
30	My voice is heard when I express my views of the quality of care in my unit.	3.30	1.25
31	When I consider safety risks, I report these risks in writing.	3.30	1.25
32	When I see a risk that could compromise the safety, I keep it to myself.	3.24	1.31
33	When I see a risk that could compromise the safety, I tell the supervisor.	3.31	1.27
34	When I see a risk that could compromise the safety, I express my concern to another employee.	3.21	1.27
35	When I see a risk that could compromise the safety, I hope that it will improve.	3.25	1.28
	overall score	3.22	1.08

**Table 3:** Results of the association between Quality Improvement Nursing Attitudes and demographic factors (*n* = 497).

Factor		Mean	*SD*	Statistical Test	*p*-Value *
Gender	Male	3.10	1.14	*t* = −3.60	<0.001
	Female	3.49	0.90		
Marital status	Single	3.40	1.03	*F* = 2.94	0.033
	Married	3.18	1.14		
	Separated	3.09	0.92		
	Widow	2.49	0.84		
Age	25–30 years old	3.01	1.17	*F* = 7.74	<0.001
	31–35 years old	3.39	0.94		
	More than 35 years old	3.41	1.05		
Level of education	Diploma	3.15	1.08	*F* = 0.52	0.596
	Bachelor’s degree	3.24	1.09		
	Other	3.31	1.18		
Experience	1–5 years	3.03	1.16	*F* = 6.515	0.002
	6–10 years	3.40	0.95		
	More than 10 years	3.11	1.13		
Hospital type	Governmental hospital	3.19	1.09	*t* = 1.40	0.16
	Private hospital	2.56	1.31		
Area of practice	Emergency Department	3.35	1.03		
	Outpatient Department	3.20	1.07	*F* = 1.31	0.25
	Medical Department	3.11	1.13		
	Surgical Department	2.95	1.13		
	ICUs	3.16	1.04		
	Artificial Kidney Unit	3.16	0.99		
	Other	2.91	1.26		
Hours worked per week	40–45 hours	3.22	1.12	*F* = 2.94	0.054
	46–50 hours	3.14	1.05		
	More than 50 hours	2.71	1.10		

* Independent sample *t*-test

## Data Availability

The datasets analyzed as part of the current study are available from the corresponding author upon reasonable request.

## List of abbreviations

QI—Quality Improvement

QINA—Quality Improvement Nursing Attitude
